# Characteristics of knowledge translation theories, models and frameworks for health technology reassessment: expert perspectives through a qualitative exploration

**DOI:** 10.1186/s12913-021-06382-8

**Published:** 2021-04-29

**Authors:** Rosmin Esmail, Fiona M. Clement, Jayna Holroyd-Leduc, Daniel J. Niven, Heather M. Hanson

**Affiliations:** 1 Department of Community Health Sciences, Cumming School of Medicine, University of Calgary, 3D10 TRW Building, 3280 Hospital Drive NW, Calgary, Alberta T2N 4Z6 Canada; 2 Alberta Health Services, Calgary, Alberta Canada; 3 O’Brien Institute for Public Health, University of Calgary, Calgary, Alberta Canada; 4 Department of Medicine, Cumming School of Medicine, University of Calgary, Calgary, Alberta Canada; 5 Hotchkiss Brain Institute, University of Calgary, Calgary, Alberta Canada; 6 Department of Critical Care Medicine, Cumming School of Medicine, University of Calgary, Calgary, Alberta Canada

**Keywords:** Health technology reassessment, Disinvestment, De-adoption, De-implementation, Theories, Models and frameworks, Knowledge translation, Implementation science

## Abstract

**Background:**

Health Technology Reassessment (HTR) is a process that systematically assesses technologies that are currently used in the health care system. The process results in four outputs: increase use or decrease use, no change, or de-adoption of a technology. Implementation of these outputs remains a challenge. The Knowledge Translation (KT) field enables to transfer/translate knowledge into practice. KT could help with implementation of HTR outputs. This study sought to identify which characteristics of KT theories, models, and frameworks could be useful, specifically for decreased use or de-adoption of a technology.

**Methods:**

A qualitative descriptive approach was used to ascertain the perspectives of international KT and HTR experts on the characteristics of KT theories, models, and frameworks for decreased use or de-adoption of a technology. One-to-one semi-structured interviews were conducted from September to December 2019. Interviews were audio recorded and transcribed verbatim. Themes and sub-themes were deduced from the data through framework analysis using five distinctive steps: familiarization, identifying an analytic framework, indexing, charting, mapping and interpretation. Themes and sub-themes were also mapped to existing KT theories, models, and frameworks.

**Results:**

Thirteen experts from Canada, United States, United Kingdom, Australia, Germany, Spain, and Sweden participated in the study. Three themes emerged that illustrated the ideal traits: principles that were foundational for HTR, levers of change, and steps for knowledge to action. Principles included evidence-based, high usability, patient-centered, and ability to apply to the micro, meso, macro levels. Levers of change were characterized as positive, neutral, or negative influences for changing behaviour for HTR. Steps for knowledge to action included: build the case for HTR, adapt research knowledge, assess context, select interventions, and assess impact. Of the KT theories, models, and frameworks that were mapped, the Consolidated Framework for Implementation Research had most of the characteristics, except ability to apply to micro, meso, macro levels.

**Conclusions:**

Characteristics that need to be considered within a KT theory, model, and framework for implementing HTR outputs have been identified. Consideration of these characteristics may guide users to select relevant KT theories, models, and frameworks to apply to HTR projects.

**Supplementary Information:**

The online version contains supplementary material available at 10.1186/s12913-021-06382-8.

## Background

Health Technology Reassessment (HTR) is an evidence-based approach that systematically reviews the clinical, social, ethical and economical effects of a technology to ensure it is being used optimally in health care [1–3]. The process results in four outputs: increase or decrease use of a technology, no change, or removal of a technology [1–3]. In recent years, HTR programs have been developed that focus on actual phases of the HTR process. A recent systematic review of HTR frameworks in seven countries identified four components: identification, prioritization, assessment, and decision dissemination strategies [3, 4]. Within the decision dissemination component, both passive (such as posting recommendations on a website) [5] and active (such as point-of-care decision support tools) [6] dissemination strategies have been proposed [7]. Despite growing interest in the HTR field, implementation challenges of its outputs continue to exist [4, 6, 8]. Implementation challenges/barriers of HTR outputs have been described previously [9]. The implementation challenges of HTR outputs have been categorized into five categories: climate and context (individuals negative attitudes, overall sense of political will, and openness to research); linkage and exchange (underlying linkage and exchange between researchers and knowledge users, policy makers and stakeholders); research evidence, a structured HTR process and resources (timelines, relevance and local applicability of research); role of researchers and HTR (the role of researchers to facilitate the transfer of research which includes views of their own role, communication, skills and packaging of research results); and role of stakeholders, knowledge users and the health system in HTR (skills and expertise) [9]. For example, in the climate and context category, one barrier is physicians may be reluctant to dismiss outmoded devices and procedures. In the role of stakeholders category, there may be a lack of understanding, resources and skills to support HTR. To address these barriers to HTR outputs, facilitators identified in the literature include strategies such as the use of champions, development of skills in the methods of HTR, development of a KT plan through the use of KT TMFs, and capacity building in KT. Therefore, KT can help address these challenges through KT strategies and interventions in the application of HTR projects.

The field of Knowledge Translation (KT) may offer a mechanism to translate these outputs into practice [9]. Knowledge Translation theories, models and frameworks (KT TMFs hereafter) have been used successfully to implement evidence into practice [10, 11]. There are a myriad of KT TMFs available to select from [12–16]. The main principle or global approach of these KT TMFs is that they all acknowledge a gap between research and application of knowledge into practice and policy. Many of these KT TMFs provide a process of how evidence and evidence-based interventions can be translated into practice [9]. Some studies have suggested the use of these KT TMFs and strategies may be useful for the implementation of HTR outputs [3, 4, 7, 9, 17]. Other studies have identified barriers and facilitators to HTR implementation and proposed strategies to address these barriers [9, 18, 19]. However, understanding which KT TMFs may be useful for the implementation of HTR outputs into practice, especially as it relates to decreased use or de-adoption of a technology, is limited [9].

A recent survey of 22 KT and HTR experts explored if full-spectrum (includes planning/design, implementation, evaluation, sustainability/scalability phases of KT) [15] KT TMFs could be suitable for HTR [20]. The survey found that ≥70% consensus was not reached on HTR suitability for any of the 16 KT TMFs that were reviewed. However, when responses to ‘yes’ and ‘partially yes’ were combined, the Consolidated Framework for Implementation Research (CFIR) [21] was considered the most suitable KT TMF by both KT and HTR experts. Moreover, the Knowledge to Action (KTA) framework [22] was selected by KT experts. HTR experts selected two additional KT TMFs: co-KT framework [23] and Plan Do Study Act (PDSA) cycle [24]. Comments provided by the experts highlighted many of the challenges related to selecting one or more KT TMFs for HTR [20]. The experts offered three key characteristics of a KT TMF that may be important to consider: practicality, guidance on implementation, and KT TMF adaptability [20]. This study emphasized that it may be difficult to find a KT TMF that addresses all of the KT considerations of the HTR process. Moreover, it may be more important to focus on specific characteristics of KT TMFs when implementing HTR outputs, in particular when decreasing use or de-adoption of technologies that are of low-value [25]. These characteristics may better inform users on how to actually decrease use or de-adopt a technology. This study aimed to determine what particular characteristics are important to consider within a KT TMF when implementing HTR outputs, specifically as it relates to decreasing use or de-adoption of a technology.

## Methods

### Study design

A qualitative descriptive approach, specifically one-to-one semi-structured interviews, was used to ascertain the perspectives of KT and HTR experts on the characteristics of KT TMFs for decreased use or de-adoption of a technology [26]. Interviews were selected as they provided an in-depth understanding of the phenomena and meaning of the key characteristics that would be critical within a KT TMF. The Consolidated Criteria for Reporting Qualitative Research Checklist (COREQ) was followed to ensure transparency, rigour, and comprehensiveness on aspects of the research team, methods, context of the study, findings, analysis, and interpretation [27] (Supplementary file 1). Ethics approval was obtained from the University of Calgary’s Conjoint Health Research Ethics Board [REB#17–0932]. Informed written consent of the participants was obtained prior to interviews. Verbal informed consent was obtained at the start of the interview using a predetermined script.

### Participant selection

In a previous study [20], we sought to survey KT and HTR international experts to determine if any KT TMFs would be suitable for HTR. KT and HTR experts were selected through purposive and snowball sampling. Names were initially derived through the KT Canada website, Health Technology Assessment international (HTAi) Disinvestment and Early Awareness Interest group, authors of relevant publications, and in consultation with other experts. A list of HTR and KT international experts was generated by country including Canada, USA, UK, Australia, and European countries (Germany, Italy, Sweden, Spain). Experts were contacted via a personalized email to verify their interest in participating in the survey study. Forty-eight KT and 31 HTR experts were invited to participate. A total of 22 experts (11 KT and 11 HTR) experts completed the survey. We used this sample of 22 experts to ask if would be interested in participating in the interviews for this study.

### Data collection

One-to-one semi-structured telephone interviews were conducted by RE from September 2019 to December 2019. Interviews ranged from 30 mins to 60 mins in duration. The interview focussed on gathering participants’ perceptions on the following: which fields (KT or HTR) they identified themselves within, general experience in using KT TMFs for HTR, experience on using specific KT TMFs on HTR, general and specific characteristics of KT TMFs for decreased use or de-adoption, barriers and facilitators for selecting KT TMFs for decreased use or de-adoption, and additional comments regarding the selection and use of KT TMFs for HTR. An interview guide was developed and tested with two members of the research team (JHL, DJN) (Supplementary file 2). Reflexive journaling and field notes were captured after each interview by RE. Anonymity was preserved by allocating all participants a code. Interviews were audio recorded and transcribed verbatim by a professional transcription company. RE also listened to the audio tapes to verify the transcription, and revise the transcripts and field notes accordingly.

### Data analysis

Data analysis occurred concurrently with data collection. The transcripts and field notes were entered into NVivo 12 Plus qualitative data analysis software to organize and code data (QSR International, MA) [28]. Framework analysis was used to analyze the data, as it allowed for a rich and in-depth analysis of the interview data through categorization of the characteristics [29–32]. Framework analysis was initially developed by Ritchie and Spencer [33] and has been further developed by others [29, 30]. It is a type of thematic analysis that has five distinctive steps: familiarization, identifying an analytic framework, indexing, charting, mapping and interpretation. Theme-based or case-based analysis, or a combination of the two, can be conducted through the development of charts [34].

#### Familiarization

RE and HMH reviewed two transcripts individually to familiarise themselves with the data and make any additional notes. Next, using an inductive approach, RE and HMH independently coded two randomly selected transcripts. Codes were generated through open coding. RE and HMH then discussed the coding together. Inter-coder reliability was found to be sufficient. This formed the basis of an initial coding structure that was applied to the rest of the transcripts that were coded by RE. Constant comparative method was used and any new codes were discussed iteratively, refined, and added to the coding structure. Code saturation was reached when no new codes emerged [35].

#### Identifying an analytic framework

Codes were clustered together into categories using an iterative process. Tree charts were developed for each category. HMH then applied these categories to one randomly selected transcript. These categories were discussed iteratively. The final list of categories and their definitions were agreed upon by RE and HMH to form the analytic framework (Supplementary file 3).

#### Indexing and charting

Indexing occurred automatically using the categories as parent codes. NVivo’s charting function was used to develop framework matrices depicting data by category and interview (case). Each matrix was exported into Microsoft Excel (Microsoft Corp., Redmond, WA, USA) for ease of readability. Interpretation and analysis between and within cases was conducted, employing the constant comparative method.

#### Mapping and interpretation

Data was analyzed thematically by reviewing the extracts within and between each case for each category. Themes were determined using the entire data set. Level one and two analyses were conducted. In level one, collated extracts for each category were read to determine if there was a coherent pattern. In level two, the characteristics were presented as over aching themes. These characteristics were reviewed to assess if they reflected the meanings evident in the data and mapping connections between categories. The themes were named and defined. Theoretical saturation was reached when no new themes emerged [36]. All decisions were made through consensus between FMC, HMH and RE. The findings were presented through a visual diagram of characteristics, themes, and illustrative quotes to exemplify each theme.

In addition, the characteristics were mapped against full-spectrum KT TMFs that had received ≥50% agreement for HTR suitability within an expert survey study [20], to determine if there was confluence between the KT TMF and the characteristics identified by the experts. The original citation that described the KT TMF was reviewed to see if the KT TMF contained any of the characteristics identified. If the KT TMF had more than 80% of the characteristics, it was also queried in NVivo to explore if the experts had identified the KT TMF and what relevant characteristics were identified.

## Results

### Participant characteristics

From September 2019 to December 2019, 13 interviews were conducted with KT (*n* = 8) and HTR experts (*n* = 5). Of the eight KT experts, three considered themselves as applied experts in KT, one as a theoretical expert in KT, and four as both applied and theoretical experts in KT. Of the five HTR experts, two considered themselves as applied experts in HTR and three as both applied and theoretical experts in HTR. Only one KT expert considered themselves as an applied expert in both KT and HTR. Whereas two HTR experts considered themselves as applied experts in both KT and HTR. Participant characteristics are presented in Table 1.

**Table 1:** Participant Characteristics (*n* = 13)

Characteristics	KT Experts (***n =*** 8)	HTR Experts (***n =*** 5)
No. of Participants	8	5
Female Sex	7	6
**Location**		
Canada	1	2
US	2	0
UK	1	1
Australia	2	1
Other	2	1
**Level of Education**		
Doctorate	7	4
Master’s	1	1
Clinical (medicine, nursing, rehab)	2	2
Years of Experience in KT field	7 to 25 years	10 to 12 years
Years of Experience in HTR field	10 to 15 years	2 to 17 years
**Self-reported activities**		
Self-Identified in KT Field	7	0
Self-identified in HTR Field	0	0
Self-identified in both	1	5

*KT* knowledge translation, *HTR* health technology reassessment

### Characteristics of a KT theory, model or framework

**Fig. 1:**
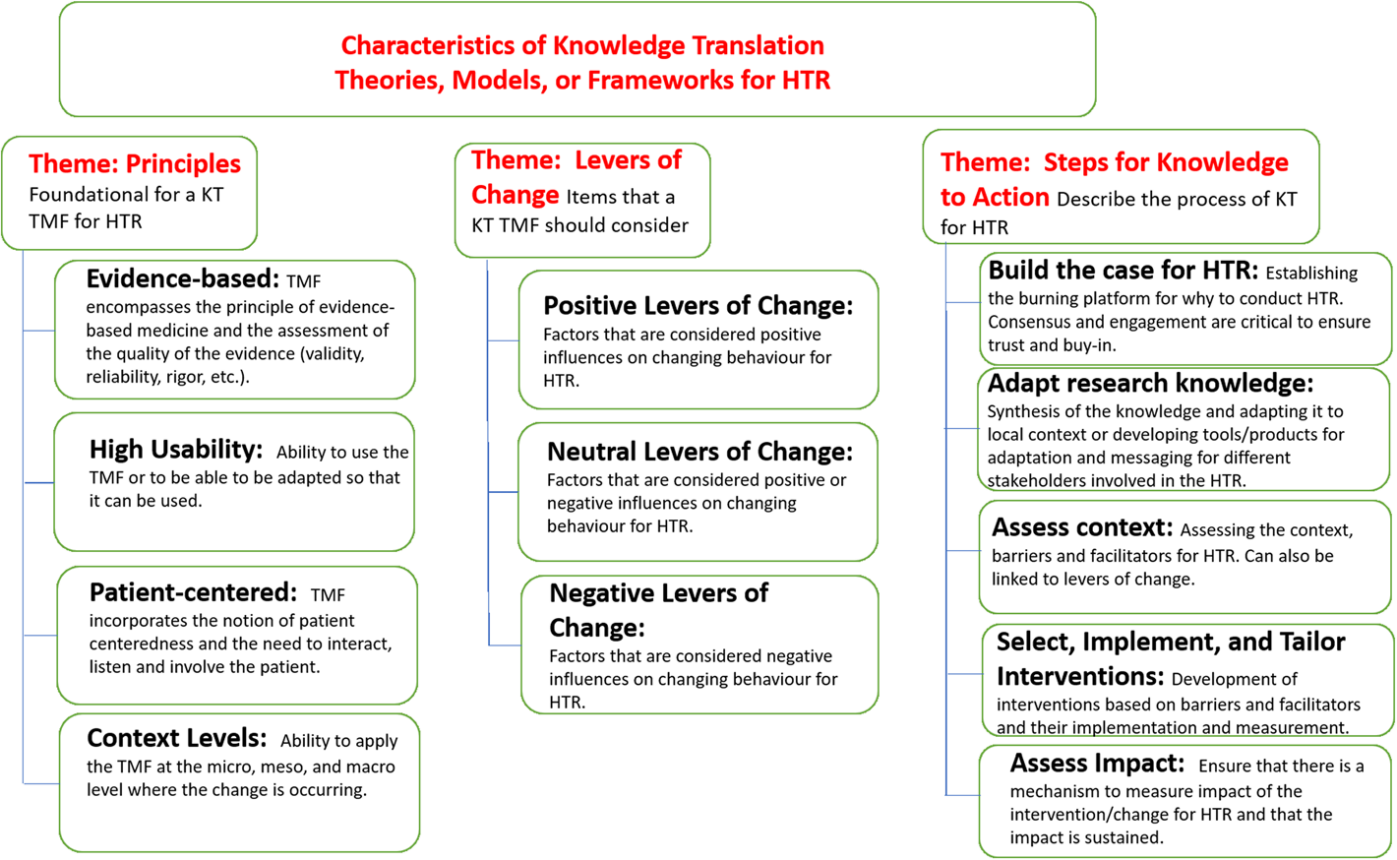
Themes and sub-themes to consider for a Knowledge Translation Theory, Model or Framework (KT TMFs) for Health Technology Reassessment (HTR)

Within the characteristics of a KT TMF, three themes emerged that illustrated the traits that a KT TMF should ideally contain to be best suited for use in HTR: principles that were foundational for HTR, levers of change, and steps for knowledge to action. Within each theme, sub-themes were identified (Fig. 1).

### Theme 1: principles that were foundational for HTR

One key theme that emerged was principles of KT TMFs that were foundational for HTR. Four principles were identified within this theme: evidence-based, high usability, patient-centered, and the ability to apply the TMF to the context levels (micro, meso, macro).

#### Principle 1: evidence-based

Participants reported that the KT TMF needed to be founded on evidence-based medicine. This entailed the qualities of face validity, transferability, generalizability and transparency. Face validity was characterized as the KT TMF was recognizable and familiar by users. The KT TMF also ‘made sense’ and could be applied in their particular setting. Transferability to other settings meant that the KT TMF could be applied to different settings and was also generalizable. Lastly, information on how the KT TMF was developed, where it has been used, guidance, tools, and instructions on the KT TMF that were available for the user reflected transparency. This was illustrated in the followingexpert:

“And then, for those who are interested, there should be transparency. All the details about the model and everything should be available.”[013]

There were some participants that felt that the guidance tools should not be too onerous to use and should be intuitive.

#### Principle 2: high usability

Participants talked about the need for the KT TMF to be applied and useful. The KT TMF should not be too ‘high level’ or ‘ivory tower’. They focussed on the need for the KT TMF to be simple, practical, and have the ability to be adapted to the particular context in which the change is occurring in. This was reflected by the following expert:

“In the context of theories it probably means that theories are more pragmatic if they short, fewer factors and if they're easier to understand, meaning that more people can understand them, independent of their disciplinary backgrounds.”[010]

Some participants noted that it may be difficult to adapt a KT TMF if it is too simple to begin with, such as the PDSA cycle. In addition, participants indicated that if it takes too much time to select a KT TMF and align it with a project, they may just select one that is easy or one they are most familiar with.

#### Principle 3: patient-centered

Participants reported that a KT TMF needed to have the ability to garner the active engagement of patients affected by the HTR process. This would also enable patients to provide ideas and strategies on how to decrease or remove a technology. Moreover, it was important not only to have patient input on the HTR process, but that patients needed to be part of the interaction and discussion during the entire reassessment process. This was exemplified by the following expert:

“We wanted to be patient-centered and so our focus was on identifying potential implementation strategies to de-implement low value care and we wanted to get patients direct input about what they thought would be a good approach for doing that. And then in a second session we invited patients and providers to work together to come up with more specific ideas. Basically, I mean we would call them the implementation strategies to de-implement specific services.”[005]

#### Principle 4: context levels

Participants conveyed that a KT TMF needed to have the ability to be applied at the micro (clinical or individual level), meso (organizational such as the hospital or regional level), and macro levels (system levels such as the provincial, state or national levels). At each context level, there may be different determinants that should be considered. The notion of vertical ‘spread’ was described by participants as important to the application of a KT TMF so that, once a reassessment is conducted at the micro level, there is ability to further spread the message and implement the findings at the meso and macro levels. Some participants felt that there could be a KT TMF that can be used for all levels (micro, meso, macro). This was illustrated by the followingexpert:

“But in terms of having an impact on the levels, one could envision either the same theory or framework being used in each of those three levels. So, for a blood transfusion, one could think a little bit about a framework that then thinks about the individual patient versus at a hospital level versus, let's say, a health system level. Right? Maybe it's the same framework that is applied at each of those levels, recognizing that, for example, the determinants, right, may be different at those three levels and could be completely different.” [019]

However, some participants thought it may be challenging to have a KT TMF that can be applied to all context levels as noted by this expert:

“I think it’s really tricky for a theory or model to be applicable on all these levels because the requirement for changing things is so difficult on the micro level compared to the macro level. I believe we need different tools, different models to work on the different context levels.” [009]

### Theme 2: levers of change

Another key theme that emerged was levers of change that would facilitate change to occur. Three types of levers of change were identified: positive, neutral and negative (Fig. 1). The positive/negative/neutral classification of levers of change was based on the lever itself and how it influenced changing behaviour within that context. For example, positive levers of change were those factors that are considered positive influences on changing behaviour for HTR such as education and training or emphasis on patient safety: neutral levers of change were those that could be considered positive or negative influences of changing behaviour such as accreditation and policy environment or cultural factors; and negative levers of change were those factors that are considered negative influences on changing behaviour for HTR such as unintended consequences or resistance factors.

#### Positive levers of change

Participants noted that within a KT TMF, positive levers of change that allowed the facilitation of HTR outputs were vital as they enabled change to happen. Training and education about the technology being decreased or de-adopted, the process of HTR, and guidance on the KT TMF that was being applied were all important considerations. Participants stated that a step-by-step way to make change happen that is self-guided would be useful. However, a ‘cookbook’ approach was not warranted, as flexibility within the KT TMF would be beneficial. Participants described using patient safety as an impetus for change to happen and providing the engagement required. Stakeholders and decision makers also needed to understand the relevance and benefits of the change. The use of both quantitative and qualitative methods to measure change was important. Lastly, alternatives to the technology being removed needed to be clearly communicated. This was exemplified by the following expert:

“In particular, in relation to de-adoption, like I mentioned earlier, the alternatives are really important. So how we quantify or evidence the alternative options available is really important for the messaging, but also for actually putting this into practice. And whether you know that’s physiotherapy or self-management or what, I think it really needs to be formally addressed. And then alternatives, as in when the resources are released, what does that mean, how do we quantify. And I think this is kind of in the messaging area, but if you’re saying we’ll release a 100 000 pounds for if we don’t do knee arthroscopy, people feel like that that’s been taken away from them rather than reduced all this harm … So you want to have a way of quantifying in a positive way, oh we’ve released 100 000 pounds and we’re reinvesting it in something else. [018]

#### Neutral levers of change

Participants talked about levers of change to be included in a KT TMF that were either positive or negative, but could also be considered to influence change for the implementation of HTR outputs. One was the use of policy or accreditation standards that compelled providers to ‘not do something’. Participants also stated to focus on a few underlying factors that could be ascertained by speaking with the stakeholders involved. These included: contextual factors (the setting in which the change is occurring), cultural factors (leadership, organizational culture, past experience with change), psychological factors (routines and habits), and technology-related factors (cost, methods used to decrease, setting, and type of indications).

#### Negative levers of change

Participants noted that within a KT TMF, elements may hinder the change to occur within a reassessment process. These negative levers of change include documentation of unwarranted variation and practice variation on different units, hospitals and between providers. This practice variation could hinder change. Subsequently, agreement on what the practice should be and convincing practitioners to change their practice would be necessary. Another was addressing the unintended consequences (positive and negative) of removing or decreasing a technology, which may impact or influence something else such as additional costs or other resources.

Participants discussed the nature of relationships between providers, the team or unit. For instance, when decreasing technology or removing technology, there may be a dynamic amongst providers, where some may want to continue to use the technology and others may not. This dynamic may drive the overuse of a particular technology by some providers, and if so, the intervention needs to target this dynamic. In addition, the notion of a power deferential between the provider, patient, and caregiver also needs to be understood. Acknowledging this power deferential and addressing it to ensure that the technology is not just being removed or decreased without engagement, and addressing patient concerns regarding technology replacement is required. Finally, understanding resistance and its causes was another lever of change. Making the case for why the technology needs to be de-adopted or decreased was imperative to address resistance. This was exemplified by the followingexpert:

“So, I think any KT theory, framework, or model needs to have within it a lens of trying to deal with confrontation or resistance from certain stakeholders, and possibly multiple stakeholders. I think that needs to be fundamental to any model.” [012]

### Theme 3: steps for knowledge to action

The third theme that emerged was that the KT TMF needed to provide steps of the knowledge to action process required for implementation of HTR outputs. There were five steps identified within this theme: i) build the case for HTR, ii) adapt research knowledge, iii) assess context, iv) select, implement and tailor interventions, and v) assess impact (Fig. 1).

#### Step 1: build the case for HTR

This step involves prioritization of HTR to justify its requirement, as not all technologies will require a reassessment. Participants noted that criteria to prioritize reassessment such as geographic variation could be used and has been outlined in other studies [3, 37]. They also added that this step focuses on identification and articulation of the problem through the synthesis of evidence on the technology and the evidence for why it should be reduced or removed. This was supported by the followingexpert:

“I mentioned the evidence is more straightforward than the actual knowledge translation, but it is kind of difficult to synthesize. And a big part of our work is sort of synthesizing the evidence in order to spread the message … But maybe something to be able to say these are the harm, benefits, strength of the evidence, evidence of variation, that kind of thing would be very helpful”. [018]

Experts also identified that buy-in from all the stakeholders impacted by the change, agreement on the problem, and engagement early on were all part of this step.

#### Step 2: adapting research knowledge

This step ensures that evidence synthesis from the ‘build the case’ step is used to develop tools and products and is customized to the local context. Participants indicated that the products (whether they are guidelines, education materials, etc.) need to be tailored to the stakeholders that are part of the reassessment process, and different products and messaging may be required. As one participant stated:

“There are many different messages that different stakeholders would want to get for that. So, we'd have to ... like all good KT ... recognize that we need different knowledge products for them. It's not about hiding things from anybody, but different people will have different interests. So, for example, let's say we're trying to decrease medical imaging. The radiologist ... There'd be something about patient safety in there. There'd be something about what the cost savings would go to. There'd may be something about ... You've got a backlog right now. We think we can clear this backlog with it. Things that, for them, would make sense. [013]

#### Step 3: assess context

This step involves evaluation of the context where the change is occurring, and identifying the barriers and enablers (determinants) to knowledge use within that context. Participants indicated that it is important not to end up with a long list of barriers, but to select from the barriers and facilitators that will have the most influence on decreased use or de-adoption of the technology. This was noted by one participant:

“Factors that are important, and then you can also use it to map responses to figure out what are the barriers, the facilitators, so to use that.”[008]

Experts noted that barriers to the reduction or removal of a technology could include cost and resource factors, behaviour and motivation factors, resistance factors, economic factors, opportunity costs, assessment of risks and unintended consequences, and personal beliefs of stakeholders. Some participants noted that the determinants would not be different from implementation of something new, but more resources would be required for decreased use or de-adoption of a technology. The levers of change identified above could also assist with understanding of barriers or facilitators to knowledge use.

#### Step 4: select, implement and tailor interventions

Participants articulated that barriers and facilitators could be used to tailor interventions. The details of the intervention needed to be explicit, so others could reproduce or adopt the intervention as needed. This was articulated by one participant:

“So, more examples of the models in action, the concrete deliverables and activities associated with implementing the models. E.G. instead of just saying, "Consult the stakeholders," be clear. Did you have 15 meetings? Did you set up a committee with the public and patient representative? What did it look like? So, if I want to do the same thing, what might I do?” [013]

Experts stated that development of measures to ensure implementation success and measurement of individual performance of the provider, unit or organization through benchmarking were key.

#### Step 5: assess impact

In this step, participants suggested the ability to evaluate the impact of the intervention to decreased use or de-adopt a technology, and that this impact was operationalized within the context. Participants noted that sustainability of the intervention to ensure decreased use or de-adoption should be considered from the beginning of the KT TMF. As one participant stated:

“I think a lot of people are using these theories and frameworks within a research project and then once the, once that project finishes, there is nothing in place to keep it embedded or sustained.” [002]

### Mapping characteristics to KT TMFs

Seven KT TMFs that had receive ≥50% agreement (yes or partially yes) from an expert survey study [20] were mapped onto the characteristics (Table 2). CFIR had the most characteristics (11/12), missing only the ability to map to the micro, meso, and macro levels [21]. This was followed by the KTA framework [22], the Quality Implementation Framework, [38] and the Healthcare Improvement Collaborative Model [39], which all had the same 10 of 12 characteristics (missing patient-centered approach and the ability to apply to the micro, meso, and macro levels). The Diffusion of Innovation [40], the co-KT framework [23], and PDSA cycle [24] had the next least number of characteristics, missing some combination of high usability, patient-centeredness, ability to apply to micro, meso, and macro levels, and levers of change.

**Table 2:** Comparison of Characteristics for Decreased Use and De-adoption within Seven Full-Spectrum Knowledge Translation Theories, Models, Frameworks (KT TMFs) that received ≥50% agreement (yes/partially yes)

KT TMF/Characteristics	Consolidated Framework for Research Implementation	Knowledge to Action Framework	Quality Implementation Framework	Healthcare Improvement Collaborative Model	Diffusion of Innovations	Co-KT framework	Plan-Do-Study-Act Cycle
**Reference**	Damschroder, 2009	Graham, 2006	Meyers, 2012	Edward, 2017	Rogers, 3rd Edition, 1983	Kitson, 2013	Deming, 1986
**Principles**							
Evidence-based	√	√	√	√	√	√	√
High Usability	√	√	√	√		√	√
Patient-Centered	√					√	
Ability to apply to micro, meso, macro levels							
**Levers**							
Positive Levers of Change	√	√	√	√	√		
Neutral Levers of Change	√	√	√	√	√		
Negative Levers of Change	√	√	√	√	√		
**Steps**							
Build the case (for HTR)	√	√	√	√	√	√	√
Adapt research knowledge	√	√	√	√	√	√	√
Assess context	√	√	√	√	√	√	√
Select, Implement, and tailor interventions	√	√	√	√	√	√	√
Assess impact	√	√	√	√	√	√	√
Total # of characteristics	11	10	10	10	9	8	7

## Discussion

### Key findings

This is the first study to interview experts in the KT and HTR fields about characteristics that need to be considered in a KT TMF for implementing HTR outputs. The study identified four principles, three levers of change, and five steps that may be important to consider when planning to reduce or remove a technology from the healthcare system.

These findings are consistent with previous research within the KT and HTR fields. In the development of a decision support tool for the selection of KT TMFs, Strifler et al. surveyed 24 KT experts from Canada, USA and Australia and found evidence, ease of use, and fit as factors that are important characteristics within a KT TMF [41]. The principle of being patient-centered has also been articulated in the HTR literature [42]. The need for stakeholder engagement as a foundational element within the HTR model has been described [3]. Meaningful and effective stakeholder engagement needs to be throughout, with engagement being authentic and early on in the process [3, 43].

A synthesis of HTR approaches and stakeholder consultation provides six questions to guide and facilitate the HTR process from a user perspective [44]. One of the six questions identifies seven levers of change to use in practice. These were drawn from the KT literature and include: clinical and/or decision-maker champions, clinical guidelines, educational initiatives, clinician reminders, audit and feedback mechanisms, incentives/disincentives, and meso/macro-level policy change. The findings from our study expands on this list of levers and further categorizing them into positive, neutral and negative.

MacKean et al. has also identified themes for moving the HTR agenda forward through shared experiences of experts from Australia, United Kingdom, and Alberta who have implemented HTR programs [42]. These themes include HTR prioritization and strong evidence for the technology being harmful, processes that are context specific, meaningful stakeholder engagement, and post-implementation monitoring and evaluation. These themes correlate to the findings from this study in terms of the steps of building the case for HTR, assessing context, and assessing impact. Ward et al., through a thematic analysis of 28 KT TMFs, found five components in their revised model on knowledge exchange: problem, context, knowledge, intervention and use [45, 46]. These are consistent with the five steps recognized by the experts as important for a KT TMF to have for decreased use or de-adoption.

The findings of this study are also similar to characteristics identified within the de-implementation literature that has largely been driven by the advent of the Choosing Wisely Campaign [47]. The Choosing Wisely lists include several recommendations in different sub-specialities focussed on reducing or removing low value care [48]. More recently, the Campaign has focussed on implementation of these recommendations with the use of effective strategies and models [49]. The Choosing Wisely Canada Implementation Research Network [50] has developed a de-implementation framework of five phases that move these recommendations into practice [17]. Phases 0 and 1 are the identification of potential areas of low-value healthcare and identification of local priorities for implementation of recommendations, which translate into ‘building the case and in this context for HTR’. Phase 2 (identification of barriers to implementing recommendations and potential interventions to overcome these) coincides with steps of ‘assessing context’ and ‘select, implement, and tailor interventions’ steps. Lastly Phase 3 (rigorous evaluations of implementation programmes) and Phase 4 (spread of effective implementation programmes) are also related to the step of ‘assessing impact’.

Of the full-spectrum KT TMFs reviewed in this study, CFIR contained most of the characteristics identified. This was also supported by the experts who had rated this KT TMF as suitable for HTR in the survey study [20]. CFIR has been identified as a highly operational framework within the implementation science field [51]. In particular, an identifiable characteristic of CFIR is its focus on the identification of 39 constructs that outline the determinants of implementation. These determinants can enable the assessment of barriers and facilitators that is part of the ‘assess context’ step required for the HTR process. The CFIR also prioritizes patient-centered approaches.

Three other frameworks, KTA, Quality Implementation Framework, and the Healthcare Improvement Collaborative Model, contained the same combination of characteristics. Moreover, experts recognized the characteristic of identified steps as distinguishable within all three KT TMFs. Although, the Quality Implementation Framework and the Healthcare Improvement Collaborative Model may be less familiar as KT TMFs, users could also apply these KT TMFs within the context of HTR.

All of the KT TMFs contained the five steps, albeit with some different labelling. This is consistent with categorization of them as full-spectrum KT TMFs that contain the four KT phases (planning/design, implementation, evaluation, sustainability/scalability). None of the KT TMFs had the ability to apply to the micro, meso, and macro levels.

The characteristics identified in this study may be useful for users to apply in the selection of a KT TMF for use in the decrease or de-adoption of a technology. Although none of the seven KT TMFs has all of the characteristics, it would be beneficial to study how the four KT TMFs with the most characteristics (CFIR, KTA, Quality Implementation Framework, and Healthcare Improvement Collaborative Model) are used in practice within the context of HTR. Case studies that focus on the KT TMF approach used, interventions developed, and lessons learned need to be further studied and shared, so that findings can be used to guide the application of KT to HTR [52–54].

### Strengths

This was the first study to specifically ask KT and HTR experts to comment on characteristics of KT TMFs that could be used for implementing HTR outputs. As such, it has advanced the literature on the application of the KT field to HTR. This study interviewed international KT and HTR experts to ensure that there was a depth and breadth of knowledge and understanding, thereby enhancing the transferability of findings. The study applied framework analysis as a systematic method to organize, categorize, analyze the data with the assistance of an experienced qualitative researcher, which enhanced the rigor and quality of the findings. Lastly, the study mapped the characteristics identified by the experts to existing KT TMFs that may be suitable for HTR.

### Limitations

Although all the experts who participated in the original survey were contacted, only 13 of the 22 experts agreed to participate in this study. However, the sample was considered acceptable to answer the research question based on achieving saturation. This was ascertained from the analysis of the data when no additional codes or themes were found after reviewing the transcript of the last interview [36]. As there were only five HTR experts and eight KT experts, the data could not be analyzed separately by KT and HTR expertise. This limited the ability for a more in-depth analysis of how the characteristics may or may not differ amongst these experts. In addition, KT experts may have limited knowledge of the HTR field given that it is a relatively new field [3]. Experts were asked to use their knowledge and understanding of KT and provide their perspective on how to apply it to the area of decreasing use and de-adoption. As each researcher’s own personal experience and perceptions may have influenced the data analysis, having RE and HMH code and categorize 23% of the transcripts in duplicate strengthened the analysis. Lastly, RE’s background in HTR and KT could have influenced the findings. However, reflexive thoughts and transparency regarding potential sources of bias were captured through journaling and field notes to minimize this potential.

## Conclusions

Implementation of HTR outputs would benefit from the application of the KT field. This study’s findings suggest that KT TMFs that present characteristics of evidence-based, high usability, are patient-centered, and application to the micro, meso, and macro levels, involve levers of change (positive, negative, and neutral), and include steps to put evidence into practice will be most useful for HTR. The application of relevant KT TMFs will enable the HTR field to move from an academic exercise to a process that ensures the optimal use of technologies within our healthcare system.

## Supplementary Information

**Additional file 1.** COREQ checklist.

**Additional file 2.** Telephone interview guide for HTR and KT experts.

**Additional file 3.** Analytic Framework.

## Data Availability

The datasets during and/or analyzed during the current study are available from the corresponding author on reasonable request.

## Abbreviations

CADTH: Canadian Agency for Drugs and Technologies in Health
CFIR: Consolidated Framework for Implementation Research
COREQ: Consolidated criteria for reporting qualitative studies
HTAi: Health Technology Assessment International
HTR: Health Technology Reassessment
KT: Knowledge Translation
KTA: Knowledge-to-Action framework
PDSA: Plan-Do-Study-Act
TMF: Theory, model, framework

## References

[1] CADTH (2019). Health Technology Reassessment: An Overview of Canadian and International Processes. (Environmental scan; no. 85).

[2] Noseworthy T, Clement FM (2012). Health technology reassessment: scope, Methdology, & language. Int J Technol Assess Health Care.

[3] Soril L, MacKean G, Noseworthy TM, Leggett LE, Clement FM (2017). Achieving Optimal Technology Use: A proposed model for health technology reassessment. SAGE Open Med.

[4] Seo H-J, Park JJ, Lee SH (2016). A systematic review on current status of health technology reassessment: insights for South Korea. Health Res Policy Syst.

[5] Garner S, Littlejohns P (2011). Disinvestment from low value clinical interventions: NICEly done?. BMJ..

[6] Henshall C, Schuller T, Mardhani-Bayne L (2012). Using health technology assessment to support optimal use of Technologies in Current Practice: the challenge of "disinvestment". Int J Technol Assess Health Care.

[7] Maloney MA, Schwartz L, O'Reilly D, Levine M (2017). Drug disinvestment frameworks: components, challenges, and solutions. Int J Technol Assess Health Care.

[8] Leggett LE, Noseworthy T, Zarrabi M, Lorenzetti D, Sutherland L, Clement F (2012). Health technology reassessment of non-drug technologies: current practices. Int J Technol Assess Health Care.

[9] Esmail R, Hanson H, Holroyd-Leduc J, Niven DJ, Clement F (2018). Knowledge translation and health technology reassessment: identifying synergy. BMC Health Serv Res.

[10] Wensing M, Grol R (2019). Knowledge translation in health: how implementation science could contribute more. BMC Med.

[11] Straus SE, Tetroe J, Graham ID. Knowledge Translation in Health Care: Moving from Evidence to Practice. 2nd ed. New York United States; 2013. p. 424.

[12] Lokker C, McKibbon KA, Colquhoun H, Hempel S (2015). A scoping review of classification schemes of interventions to promote and integrate evidence into practice in healthcare. Implement Sci.

[13] Milat AJ, Li B (2017). Narrative review of frameworks for translating research evidence into policy and practice. Public Health Res Pract.

[14] Tabak RG, Khoong EC, Chambers DA, Brownson RC (2012). Bridging research and practice: models for dissemination and implementation research. Am J Prev Med.

[15] Strifler L, Cardoso R, McGowan J, Cogo E, Nincic V, Khan PA, Scott A, Ghassemi M, MacDonald H, Lai Y, Treister V, Tricco AC, Straus SE (2018). Scoping review identifies number of knowledge translation theories, models and frameworks with limited use. J Clin Epidemiol.

[16] Esmail R, Hanson HM, Holroyd-Leduc J, Brown S, Strifler L, Straus SE, Niven DJ, Clement FM (2020). A scoping review of full-spectrum knowledge translation theories, models, and frameworks. Implement Sci.

[17] Grimshaw JM, Patey AM, Kirkham KR, Hall A, Dowling SK, Rodondi N, et al. De-implementing wisely: developing the evidence base to reduce low-value care. BMJ Qual Saf. 2020;29(5):409–17. 10.1136/bmjqs-2019-010060. PMC7229903 32029572

[18] Soril LJJ, Noseworthy TW, Stelfox HT, Zygun DA, Clement FM (2019). Facilitators of and barriers to adopting a restrictive red blood cell transfusion practice: a population-based cross-sectional survey. CMAJ open.

[19] Mayer J, Nachtnebel A (2015). Disinvesting from ineffective technologies: lessons learned from current programs. Int J Technol Assess Health Care.

[20] Esmail R. Understanding the Relationship Between Health Technology Reassessment and Knowledge Translation. 2020. University of Calgary, PhD dissertation. https://prism.ucalgary.ca/handle/1880/112700.

[21] Damschroder LJ, Aron DC, Keith RE, Kirsh SR, Alexander JA, Lowery JC (2009). Fostering implementation of health services research findings into practice: a consolidated framework for advancing implementation science. Implement Sci.

[22] Graham ID, Logan J, Harrison MB, Straus SE, Tetroe J, Caswell W, Robinson N (2006). Lost in knowledge translation: time for a map?. J Contin Educ Heal Prof.

[23] Kitson A, Powell K, Hoon E, Newbury J, Wilson A, Beilby J (2013). Knowledge translation within a population health study: how do you do it?. Implement Sci.

[24] Deming W. Plan-do-study-act (PDSA) cycles. 1986. [Available from: https://deming.org/explore/pdsa/].

[25] Elshaug AG, McWilliams JM, Landon BE (2013). The value of low-value lists. JAMA..

[26] Neergaard MA, Olesen F, Andersen RS, Sondergaard J (2009). Qualitative description – the poor cousin of health research?. BMC Med Res Methodol.

[27] Tong A, Sainsbury P, Craig J (2007). Consolidated criteria for reporting qualitative research (COREQ): a 32-item checklist for interviews and focus groups. Int J Qual Health Care.

[28] QSR International. NVivo [12 Plus ed.]. 2018. Available from [https://www.qsrinternational.com/nvivo-qualitative-data-analysis-software/about/nvivo].

[29] Ward DJ, Furber C, Tierney S, Swallow V (2013). Using framework analysis in nursing research: a worked example. J Adv Nurs.

[30] Gale NK, Heath G, Cameron E, Rashid S, Redwood S (2013). Using the framework method for the analysis of qualitative data in multi-disciplinary health research. BMC Med Res Methodol.

[31] Armson H, Lockyer JM, Zetkulic M, Könings KD, Sargeant J (2019). Identifying coaching skills to improve feedback use in postgraduate medical education. Med Educ.

[32] Roze des Ordons A, Cheng A, Gaudet J, Downar J, Lockyer J. Adapting feedback to individual residents: an examination of preceptor challenges and approaches. J Grad Med Educ. 2018;10(2):168–75. 10.4300/JGME-D-17-00590.1. 10.4300/JGME-D-17-00590.1 PMC5901796 29686756

[33] Ritchie J, Spencer L (1994). Qualitative analysis for applied policy research.

[34] Rabiee F (2004). Focus-group interview and data analysis. Proc Nutr Soc.

[35] Kerr C, Nixon A, Wild D (2010). Assessing and demonstrating data saturation in qualitative inquiry supporting patient-reported outcomes research. Expert Rev Pharmacoecon Outcomes Res.

[36] Hennink MM, Kaiser BN, Marconi VC (2017). Code saturation versus meaning saturation: how many interviews are enough?. Qual Health Res.

[37] Paprica PA, Culyer AJ, Elshaug AG, Peffer J, Sandoval GA (2015). From talk to action: policy stakeholders, appropriateness, and selective disinvestment. Int J Technol Assess Health Care.

[38] Meyers DC, Durlak JA, Wandersman A (2012). The quality implementation framework: a synthesis of critical steps in the implementation process. Am J Community Psychol.

[39] Edward K-L, Walker K, Duff J (2017). A multi-state, multi-site, multi-sector healthcare improvement model: implementing evidence for practice. Int J Qual Health Care.

[40] Rogers EM (1983). The innovation-decision process.

[41] Strifler L, Barnsley JM, Hillmer M, Straus SE (2020). Identifying and selecting implementation theories, models and frameworks: a qualitative study to inform the development of a decision support tool. BMC Med Inform Decis Mak.

[42] MacKean G, Noseworthy T, Elshaug AG, Leggett L, Littlejohns P, Berezanski J, Clement F (2013). Health technology reassessment: the art of the possible. Int J Technol Assess Health Care.

[43] Sevick K, Soril LJJ, MacKean G, Noseworthy TW, Clement FM. Unpacking early experiences with health technology reassessment in a complex healthcare system. Int J Healthc Manag. 2017:1–7.

[44] Soril LJJ, Niven DJ, Esmail R, Noseworthy TW, Clement FM (2018). Untangling, unbundling, and moving forward: framing health technology reassessment in the changing conceptual landscape. Int J Technol Assess Health Care.

[45] Ward V, Hamer S, House A (2009). Developing a framework for transferring knowledge into action: a thematic analysis of the literature. J Health Serv Res Policy.

[46] Ward V, Smith S, Hamer S, House A (2012). Exploring knowledge exchange: a useful framework for practice and policy. Soc Sci Med.

[47] Levinson W, Kallewaard M, Bhatia RS, Wolfson D, Shortt S, Kerr EA (2015). ‘Choosing wisely’: a growing international campaign. BMJ Qual Saf.

[48] Choosing Wisely Canada. 2017 [Available from: https://choosingwiselycanada.org/].

[49] van Bodegom-Vos L, Davidoff F (2017). Marang-van de Mheen PJ. Implementation and de-implementation: two sides of the same coin?. BMJ Qual Saf.

[50] Choosing Wisely Implementation Research Network. 2020 [Available from: https://choosingwiselycanada.org/implementation-research-network].

[51] Birken SA, Powell BJ, Presseau J, Kirk MA, Lorencatto F, Gould NJ, Shea CM, Weiner BJ, Francis JJ, Yu Y, Haines E, Damschroder LJ (2017). Combined use of the consolidated framework for implementation research (CFIR) and the theoretical domains framework (TDF): a systematic review. Implement Sci.

[52] Niven DJ, Mrklas KJ, Holodinsky JK, Straus SE, Hemmelgarn BR, Jeffs LP, Stelfox HT (2015). Towards understanding the de-adoption of low-value clinical practices: a scoping review. BMC Med.

[53] Polisena J, Clifford T, Elshaug AG, Mitton C, Russell E, Skidmore B (2013). Case studies that illustrate disinvestment and resource allocation decision-making processes in health care: a systematic review. Int J Technol Assess Health Care.

[54] Hollingworth W, Rooshenas L, Busby J, Hine CE, Badrinath P, Whiting PF, Moore THM, Owen-Smith A, Sterne JAC, Jones HE, Beynon C, Donovan JL. Using clinical practice variations as a method for commissioners and clinicians to identify and prioritise opportunities for disinvestment in health care: a cross-sectional study, systematic reviews and qualitative study. Southampton: NIHR Journals Library; 2015. https://pubmed.ncbi.nlm.nih.gov/25879119/. 25879119

