# Supplementary material for: Characteristics of knowledge translation theories, models and frameworks for health technology reassessment: expert perspectives through a qualitative exploration
Source: BMC Health Serv Res. 2021 Apr 29;21:401. doi: 10.1186/s12913-021-06382-8 (PMC8082625; doi:10.1186/s12913-021-06382-8)
Supplement: Supplementary file 1 — Additional file 1. COREQ checklist. [file 12913_2021_6382_MOESM1_ESM.docx]

**Supplementary File 1: Consolidated criteria for reporting qualitative studies (COREQ): 32-item checklist**

| **No. Item** | **Guide questions/description** | **Reported on Page #** |
| --- | --- | --- |
| **Domain 1: Research team and reﬂexivity** |  |  |
| *Personal Characteristics* |  |  |
| 1. Interviewer/facilitator | Rosmin Esmail | Methods |
| 2. Credentials | BSc, MSc, PhD Candidate | Title Page |
| 3. Occupation | PhD student/Provincial Trauma Epidemiologist for Alberta Health Services | N/A |
| 4. Gender | Female | N/A |
| 5. Experience and training | Rosmin Esmail is a PhD Candidate. She conducted this study as part of her PhD. She has training as an epidemiologist. Through her PhD she has received training in health services research and methodology. She also received formal training in qualitative methods. | N/A |
| *Relationship with participants* |  |  |
| 6. Relationship established | Rosmin Esmail contacted the participants by email to determine if they were able to participate and schedule the interview. Some of the participants were known to Rosmin Esmail. | N/A |
| 7. Participant knowledge of the interviewer | Participants were aware of the purpose of the study, its rationale, and that it was being conducted as part of Rosmin Esmail’s PhD thesis. | Methods |
| 8. Interviewer characteristics | Rosmin Esmail disclosed her role as a PhD candidate at the University of Calgary and as the primary researcher for this project. | Methods |
| **Domain 2: study design** |  |  |
| *Theoretical framework* |  |  |
| 9. Methodological orientation and Theory | Framework analysis. | Methods |
| *Participant selection* |  |  |
| 10. Sampling | Participants were selected through purposeful sampling. During the recruitment, we were mindful to ensure representatives from different countries, knowledge and expertise, and roles. It was this group of participants who were also asked to participate in the interviews. | Methods |
| 11. Method of approach | Participants were approached via a personalized email. | Methods |
| 12. Sample size | 13 participants. | Results |
| 13. Non-participation | 9 participants. Four participants refused to participate and 5 did not respond. | Methods |
| *Setting* |  |  |
| 14. Setting of data collection | Phone interviews were conducted from Rosmin Esmail’s home or office at Foothills Medical Center. Participants conducted the interviews from their preferred location. | Methods |
| 15. Presence of non-participants | No. | Results |
| 16. Description of sample | See Table 1. Most of the participants had a doctorate degree. They had a range of experience in knowledge translation and/or health technology reassessment. | Results |
| *Data collection* |  |  |
| 17. Interview guide | See Supplementary File 2. A semi-structured interview guide was developed and tested with two member of the research team, both of whom were clinicians. The interview questions were informed by the open-ended comments from the previous study that surveyed the experts. | Methods |
| 18. Repeat interviews | No. | N/A |
| 19. Audio/visual recording | The interviews were audio-recorded and transcribed verbatim by a professional transcription company. Rosmin Esmail also listened to the audio tapes to verify the transcription. | Methods |
| 20. Field notes | Field notes were made by Rosmin Esmail during and after each interview. These notes were referred to during the data analysis and interpretation. | Methods |
| 21. Duration | Interview duration was between 30 to 60 minutes. | Methods |
| 22. Data saturation | Code saturation was reached when no new codes emerged. Any new codes that were derived from the final interview transcript were checked with the existing coding structure to determine if they were new codes or had been already derived. Thematic saturation was reached when no new themes emerged. | Methods |
| 23. Transcripts returned | No. | N/A |
|  |  |  |
| Domain 3: analysis and ﬁndings |  |  |
| Data analysis |  |  |
| 24. Number of data coders | Data were inductively coded by Rosmin Esmail. 15% of the transcripts were coded independently by a second investigator. Categories for the analytic framework were generated by Rosmin Esmail. See Supplementary File 3. A second investigator applied these categories independently to one randomly selected transcript and these were discussed iteratively. | Methods |
| 25. Description of the coding tree | A coding tree was developed. A description of the coding tree was not provided but will be made available upon request. | Methods |
| 26. Derivation of themes | Themes and sub-themes were derived from the data. | Methods |
| 27. Software | NVivo 12 Plus qualitative data analysis software (QSR International, Cambridge, MA) was used to organize and code the data. | NVivo |
| 28. Participant checking | No. | N/A |
| Reporting |  |  |
| 29. Quotations presented | The direct quotes from participants were provided in the transcript to support the themes. Participant ID numbers were provided for each quote. | Results |
| 30. Data and ﬁndings consistent | There was consistency between the data presented and the ﬁndings through the codes, tree chart, analytic framework and development of themes. | Results/Discussion |
| 31. Clarity of major themes | See Figure 1. The major themes that depicted characteristics of KT TMFs included: principles or foundational for a KT TMF for HTR, levers of change that a KT TMF should consider, and steps that describe the process of KT for HTR. | Results/Discussion |
| 32. Clarity of minor themes | See Figure 1. The major themes were further categorized into sub-themes. For example, the principles theme, four sub-themes emerged: evidence-based, high usability, patient-centered, and context levels. | Results/Discussion |
