# Supplementary material for: Characteristics of knowledge translation theories, models and frameworks for health technology reassessment: expert perspectives through a qualitative exploration
Source: BMC Health Serv Res. 2021 Apr 29;21:401. doi: 10.1186/s12913-021-06382-8 (PMC8082625; doi:10.1186/s12913-021-06382-8)
Supplement: Supplementary file 2 — Additional file 2. Telephone interview guide for HTR and KT experts. [file 12913_2021_6382_MOESM2_ESM.docx]

**Supplementary File 2: Telephone Interview Guide for Semi-Structured Interviews with HTR/KT Experts**

**Study ID: _______**

**INTRODUCTION**

Thanks for agreeing to speak with me. I wanted to re-iterate the purpose of this study. I am conducting a study on understanding the relationship between health technology reassessment (HTR) and knowledge translation (KT). Understanding the relationship between HTR outputs and KT is important as it provides a mechanism for determining what and how the two are linked and if KT can play a role in the advancement of HTR. If you recall in the first round you critically reviewed full-spectrum KT theories, models, or frameworks.

Analysis of the data from round one of the modified Delphi process indicates that ≥ 70% consensus was not reached on HTR suitability for any of the 16 KT theories, models and frameworks (TMFs). The comments that were provided in the survey by you were invaluable and highlight the challenges in selecting one or more KT TMFs for HTR. This process has also emphasized that it may be difficult to find a KT TMF that addresses all of the KT considerations of the HTR process.

As a result, for round two of the modified Delphi process, we would like to further investigate the ***key characteristics*** of a KT TMF for HTR that maybe important to consider. In particular, we would like to examine of the KT TMFs that you reviewed, what were the elements, attributes, constructs that you think would influence and demonstrate an important role within the process of HTR.

As an expert on *<KT or HTR>*, meeting with you is important to provide your input. The interview will take about 30 to 60 mins. Do you have time available today? *<If response is no then offer to reschedule>.*

The interview will be recorded and transcribed. Is that okay? <If response is no then indicate that I will be taking notes>. The information you provide to me today will be kept strictly confidential and your identity will be protected. In the analysis, I will not be identifying the person or site. Please keep in mind that you are able to withdraw at any time during the interview process.

Do you have any questions about this interview or study before we begin?

***Opening Questions***

Which field do you identify yourself in Knowledge Translation (KT), health technology reassessment (HTR) or both?

How long have you been in this field/doing research in this field/both of these fields?

1. Do you consider yourself an applied KT/HTR expert or do you consider yourself more an expert on the development of KT TMFs/the development of the HTR process?

***General* Experience in using KT theories, models, frameworks for HTR**

As you are aware, health technology reassessment is about reassessing technologies (drugs, medical devices, diagnostic tests, i.e.) that are currently being used in the system. In the survey, you had an opportunity to review 16 KT theories, models, and frameworks to assess their suitability for HTR. Now I’d like you to think about these KT theories, models or frameworks in general within the context of HTR.

1. What has been your experience in general in using KT theories, models or frameworks for HTR projects in particular?

***If the respondent says they have experience in using KT theories, models or frameworks for HTR go to question #5***

***If the respondent says they have no experience or minimal experience in using KT theories, models, or frameworks for HTR go to question #8***

**Experience in using *specific* KT theories, models, frameworks for HTR**

Now we will focus on the *specific* KT theories, models or frameworks within the context of health technology reassessment (HTR).

1. Please describe your experience in using *specific* KT theories, models or frameworks used within the context of HTR?

***If the respondent says they have experience in using specific KT theories, models or frameworks for HTR go to question #6***

***If the respondent says they have no experience or minimal experience in using specific KT theories, models, or frameworks for HTR go to question #8***

1. Why did you select these specific KT theories, models, or frameworks?
2. In your view, what made the KT theory, model or framework appropriate to use in HTR? <go to question #8>

***General Characteristics* of KT theories, models, frameworks for Decrease Use or De-adoption**

1. In HTR there are four outputs (increase use, no change, decrease use or de-adoption). Today we are focussing on the outputs of decrease use and de-adoption. When using a KT theory, model or framework, what are the particular characteristics <give example if needed: such as ease of use or adaptability> that are important to consider specifically for the outputs of decreasing use or de-adopting a technology? *Probe:* why did you consider these as important?

***Specific Characteristics* of KT theories, models, frameworks for Decrease Use or De-adoption**

Now we will focus on the *specific characteristics* of KT theories, models, or frameworks that you mentioned in the previous question.

1. What does the <particular characteristic(s)> mean to you? *Probe:* how would the particular characteristic(s) be demonstrated to you?

There were several *specific* characteristics that were highlighted in the survey responses that I received for the KT theories, models, and frameworks that were reviewed. These were considered as important for the HTR process in round one.

1. One of them was **pragmatic**. What does **pragmatic** mean to you in the context of a TMF? *Probes:* How would pragmatic be demonstrated in a TMF? What does comprehensive or process-oriented mean to you?
2. Another ***characteristic*** was that a TMF should provide ***guidance in implementation*** (how to do the work) of its outputs. What does implementation mean to you in the context of a TMF? *Probe:* How would guidance on implementation be demonstrated in a TMF?
3. Another ***characteristic*** was that a TMF should have ***a strong fit or adaptability to the HTR process.*** What does strong fit/adaptability to HTR mean to you in the context of a TMF? *Probe:* How would strong fit/adaptability be demonstrated in a TMF?
4. Another TMF ***characteristic*** that was highlighted was the need for the TMF ***to engage stakeholders***. What does that mean to you?  *Probes:* How would the TMF involve stakeholders? Who would these stakeholders be?
5. ***Understanding determinants*** ***(for example barriers or enablers that might influence an implementation outcome*** **such as knowledge or intention)** that would facilitate implementation was another ***characteristic***. What are your thoughts on the determinants for developing interventions/strategies for the HTR process? *Probes:*  What would these determinants be? How would their inter-relationships be expressed?
6. Another TMF ***characteristic*** was the ***ability to apply the TMF*** ***at the micro, meso, macro levels.*** What do these levels mean to you? *Probes:* How would you define these levels? What about the ability to tailor the TMF at these levels? What does this mean to you?

**Application of KT theories, models, frameworks for Decrease Use or De-adoption**

Now we will focus on the *application* of KT theories, models or frameworks within the context of the decrease use and de-adoption of the HTR process.

1. In general, what approaches or decision-making processes would you use to go about identifying and selecting a KT theory, model, or framework for the outputs of decrease use or de-adoption?
2. In general, how would you go about applying a KT theory, model, or framework for the outputs of decrease use or de-adoption?

**Barriers and Facilitators**

1. Overall, what do you think are the greatest challenges to identifying, selecting and using KT theories, models or frameworks for decrease use or de-adoption? *Probes:* How and why are these factors major issues or barriers to success? How do you think they could be addressed?
2. What factors do you think facilitate the identification, selection and use of KT theories, models, and frameworks for decrease use or de-adoption? *Probe:* How and why are these factors facilitators to success?

**Additional comments and wrap-up**

1. Before we wrap up, do you have any additional comments to add regarding the selection and use of KT theories, models or frameworks for HTR?
2. Is there anything else you feel we did not cover that I need to know?

**Thank you for your time.**
