# Supplementary material for: Characteristics of knowledge translation theories, models and frameworks for health technology reassessment: expert perspectives through a qualitative exploration
Source: BMC Health Serv Res. 2021 Apr 29;21:401. doi: 10.1186/s12913-021-06382-8 (PMC8082625; doi:10.1186/s12913-021-06382-8)
Supplement: Supplementary file 3 — Additional file 3. Analytic Framework. [file 12913_2021_6382_MOESM3_ESM.docx]

**Supplementary File 3: Analytic Framework**

| **Category** | **Demographic-related** | **Principles** | **Levers of Change** | **Steps** | **Factors associated with the use** | **Other Clarification** | **Other Tangential** |
| --- | --- | --- | --- | --- | --- | --- | --- |
| **Operational Definition of Category** | Codes (text) that are related to the participant characteristics. | Codes (text) that are that TMF should have as necessary and foundational for HTR. | Codes (text) that the TMF should consider for HTR. | Codes (text) that are the steps in a KT TMF that describe the process of KT for HTR. | Codes (text) that are related to using KT TMFs for HTR, their purpose, their selection, challenges, facilitators to enable use. | Codes (text) that seek clarification of concepts. | Codes (text) that are peripheral to the research question. They do not address the research question specifically. |

KT=knowledge translation; HTR=health technology reassessment; TMFs=theories, models, frameworks
